# Voting in a Pandemic: Explaining Variation in Support for Absentee Ballots for All

**DOI:** 10.1017/S1743923X20000641

**Published:** 2020-12

**Authors:** Melanie Sayuri Dominguez, Edward D. Vargas, Gabriel R. Sanchez

**Affiliations:** 1 University of New Mexico; 2 Arizona State University; 3 University of New Mexico

**Keywords:** COVID-19, voting by mail, gender

## Abstract

The COVID-19 pandemic has fundamentally changed all aspects of American life, including, for many, how we vote. We explore the question of who supports unrestricted absentee ballots during a pandemic. We argue that women are more likely to support absentee ballots because they allow for greater flexibility and minimize the potential for exposure. We test this theory using the National Panel Study of COVID-19 (*n* = 1,892), which asked respondents about their preferences for absentee ballots, their worry about the coronavirus, and their household composition. Using multinomial logistic regressions, we find that women are more likely to support allowing absentee ballots compared with more restrictive voting options and are more likely to say they support absentee ballots for all if they know someone who has contracted COVID-19. The policy implications for these findings are discussed along with other sociodemographic indicators in our analysis.

Countries around the world continue to fight the spread of COVID-19 by mandating or strongly encouraging people to practice social distancing, work from home when possible, and wear a mask in public. This entails not only changes in behavior by people but also adjustments by governments to minimize social interactions among their citizens. One of the most contentious steps that states have considered during this period is how to administer primary elections. While many states delayed their primaries or rescheduled them to make mail-based voting more accessible to voters, others (most prominently Texas) maintained more restrictive in-person voting procedures. Little work has explored how the electorate feels about voting at a time when doing so is potentially hazardous to one's health.

Who among the electorate supports mail voting in light of the COVID-19 pandemic? We argue that women are, in general, more likely to support mail voting primarily because it allows for greater flexibility and because they may be more concerned with the potential spread of the virus and the impact it may have on their families.

## MAIL VOTING IN THE UNITED STATES

Mail voting looks different across the United States, with some states and counties being very restrictive about who can request an absentee ballot, others not requiring an excuse, and some having universal mail voting. Given this variation, scholars who explore the effect of voting by mail on turnout and participation have come to mixed conclusions.

Some argue that participation in mail voting will resemble voting in polling places (Magleby 1987). Others argue that mail voting increases participation in the aggregate, mostly affecting people who are low-participating registrants (Gerber, Huber, and Hill 2013). However, in the context of this public health crisis, it is very likely that requiring in-person voting will decrease turnout (Barreto et al. 2020). There does not appear to be any doubt that moving away from in-person voting will prevent the spread of the coronavirus, particularly in November, when public health experts predict a spike. Several studies have shown that the virus does not survive on paper or cardboard for very long, making mail voting a safe alternative (DeWitt, St. Maurice, and Rios 2020).

Despite the positives of mail voting during a pandemic, some worry about possible negative consequences, namely, the increased risk of voter fraud or the change in voting procedure favoring one party over another. However, studies have found that the risk of voter fraud is not substantiated (Barreto et al. 2020) and that universal vote by mail does not appear to have a partisan bias (Thompson et al. 2020).

## WOMEN'S SUPPORT FOR VOTE BY MAIL

Past research into attitudes toward vote by mail has shown that women tend to support mail voting at higher levels than men, possibly because of the added flexibility of this type of voting for women who juggle multiple responsibilities (Southwell 2004, 2007). This flexibility is helpful in two ways: women tend to be hourly wage employees at higher levels than men, meaning that voting may cut into work time and pay. Second, especially during this pandemic, women report increasing difficulty in balancing work and life, which should lead women to be likelier to report wanting a more flexible means of voting.^1^

We further hypothesize that having someone in their network who has contracted COVID-19 should lead women to be more likely to support universal absentee ballots considering the greater risk associated with in-person voting. Studies have found that direct/personal and meaningful experience may alter people's attitudes toward green politics and racial and gender biases (e.g., Rudman, McLean, and Bunzl 2013).^2^ Direct experience with COVID-19 operates similarly to encourage those who were less likely to support preventive measures before to change their minds. We argue that this effect is different for men and women, because women are primary caregivers for children, parents, and family members with special needs at higher rates than men^3^ and therefore may be less likely to want to put these family members unnecessarily at risk by having to go to a polling place to vote.

These differences in the lived experiences of men and women, coupled with a literature that has identified differences in policy and political preferences between men and women (Barnes 2012; Inglehart and Norris 2000; Swers 1998), leads us to the following hypotheses:

**H_1_**: Women are more likely to support universal absentee ballots for the November 2020 election.

**H_2_**: Women are more likely to support universal absentee ballots if they have someone in their network who has had COVID-19.

## DATA AND METHODS

To test our theory, we use survey data from the National Panel Study of COVID-19, a multi-wave panel survey conducted in March, April, and June 2020.^4^ Respondents were asked several behavioral and attitudinal questions surrounding the COVID-19 pandemic. The second wave of this nationally representative survey was conducted between April 14 and 21, 2020 (*n* = 1,892). It included the following question: “Do you think states should allow anyone to request an absentee ballot and vote by mail, or should states be allowed to restrict mail ballots to just the elderly or military who are overseas?” Among the respondents, 13% said that they believed absentee ballots should be restricted to the elderly and military abroad, 36% responded that it depends, and 51% answered that anyone should be allowed to request one. When we disaggregate by gender, we find support for our hypothesis: men are less likely than women to support absentee ballots for everyone (47% compared with 54% for women). Additionally, men are more likely than women to think that absentee ballots should be restricted to the elderly and overseas military (16% compared with 10%).

We employ multinomial logistic regression, using support for restricting absentee ballots only for military and the elderly as the base category. To assess our theory regarding higher concerns about the virus among women, we included variables asking how worried respondents were that they or their loved ones would get the virus, as well as whether they already had people who had gotten sick from the virus in their network (this included themselves, immediate family, and work colleagues). We further controlled for variables that have been found to affect political attitudes, including partisanship, self-assessment on the political ideology scale, age, income, education, employment status, and household composition.

## RESULTS AND DISCUSSION

Our results (Table 1) are separated into three models: the full model, male respondents, and female respondents^5^. Overall, we find support for our hypotheses. In our full regression model, we see that women are 1.26 times likelier than men to support unrestricted absentee voting.

**Table 1:** (abbreviated). Multinomial logistic regression coefficients for the probability for support for absentee ballots using restrict as the baseline comparison using the National Panel Study of COVID-19

	*Full Model*		*Men*		*Women*	
	Allow	Depends	Allow	Depends	Allow	Depends
Variables	Odds Ratio	Odds Ratio	Odds Ratio	Odds Ratio	Odds Ratio	Odds Ratio
Female	1.258*	0.499***				
	(0.121)	(0.178)				
Corona worry	1.033	0.612***	1.075	0.535**	0.990	0.740
	(0.131)	(0.177)	(0.209)	(0.260)	(0.166)	(0.235)
Corona network risk	1.509**	0.828	1.360	0.649	1.655**	0.863
Reference: Republican						
Democrat	1.546***	0.557**	1.122	0.426**	2.082***	0.715
	(0.169)	(0.262)	(0.253)	(0.381)	(0.215)	(0.367)
Independent	1.121	1.106	0.822	1.137	1.425	0.910
	(0.171)	(0.225)	(0.264)	(0.326)	(0.217)	(0.310)
Political ideology (Lib.– Cons.)	0.735***	1.529***	0.730***	1.617***	0.720***	1.311**
	(0.063)	(0.094)	(0.093)	(0.131)	(0.081)	(0.135)
Reference category: Single no kids						
Single with kids	0.816	1.019	0.631	1.402	0.908	0.787
	(0.285)	(0.661)	(0.501)	(0.856)	(0.354)	(1.139)
Married no kids	0.881	1.374	1.150	1.332	0.674	1.834
	(0.203)	(0.318)	(0.302)	(0.426)	(0.267)	(0.486)
Married with kids	0.637**	1.030	0.582*	1.184	0.738	1.426
	(0.204)	(0.334)	(0.300)	(0.436)	(0.281)	(0.553)
Divorced no kids	0.652*	0.979	0.770	0.861	0.568**	1.239
	(0.231)	(0.325)	(0.375)	(0.445)	(0.287)	(0.495)
All else	0.727*	1.190	0.838	1.631	0.634*	1.043
	(0.187)	(0.313)	(0.289)	(0.409)	(0.247)	(0.477)
Observations	1,892	1,892	776	776	1,116	1,116

*Note:* Robust standard errors in parentheses, using national weights, adjusting for race/ethnicity, education, age, income, and employment status.

*** *p* < .01; ** *p* < .05; * *p* < .1.

For the separate models, factors that we would expect to affect support for universal absentee ballots are significant only for women. For example, having a person in one's network infected who has contracted the coronavirus increases women's support for universal absentee ballots. Specifically, women with someone in their network are 65.5% likelier to support universal absentee ballots than women who do not. This may be because having someone in one's network may make women more aware of the importance of preventive measures. Interestingly, neither being worried about the coronavirus nor having an infected person in one's network seems to affect men's likelihood of supporting universal absentee ballots. On the contrary, being worried about the coronavirus decreases the likelihood that men say that “it depends” in response to the question of support for absentee ballots.

While media attention has focused on the partisan divide of supporting absentee ballots,^6^ this effect appears to be driven by Democratic women, not men. Democratic women are twice as likely as non-Democratic women to support universal absentee ballots. Again, in the opposite direction as expected, Democratic men are less likely to say “it depends” compared with fully restricting absentee ballots. Both conservative women and men are less likely to support allowing universal absentee ballots.

Family composition also matters in whether men and women support universal absentee ballots. Men who are married with children are less likely to support universal absentee ballots. Similarly, divorced women without children are also less likely to support universal absentee ballots. Most of our control variables also operate in the direction as expected, at least for women, with higher levels of education, older age, and more family income leading to likelier support for universal absentee ballots.

## CONCLUSION

We explored who across the population supports universal absentee ballots at a critical time in American history to understand this question. We find support for our hypotheses that women are more likely to support universal absentee ballots. In light of the coronavirus pandemic, we also find that having someone in one's network who has been infected with the virus leads women to be more likely to support universal absentee ballots.

It is important for policy makers to consider and recognize the views of the public during extraordinary times like these when voting could harm voters. This means that it is eminent, especially during a global pandemic, to make voting an easier and more accommodating process that simplifies voting for both men and women.

## Footnotes

1. See, e.g., Patricia Cohen and Tiffany Hsu, “Pandemic Could Scar a Generation of Working Mothers,” New York Times, June 3, 2020, https://www.nytimes.com/2020/06/03/business/economy/coronavirus-working-women.html (accessed September 9, 2020).

2. This study is based on experiencing extreme weather and support for green politicians. While the focus of that study is changes in implicit attitudes toward green politicians after hurricane experience, we believe the logic of the argument applies very similarly to knowing someone with COVID-19.

3. Based on the Centers for Disease Control and Prevention's Behavioral Risk Factor Surveillance System data from 2015–16.

4. These data were collected using Lucid Marketplace, which connects researchers with respondents. More information can be found at https://luc.id/. We added appropriate weights to the data for it to be appropriate of the American population.

5. The full model with all of the control variables can be found in the appendix in the supplementary material online.

6. Pew Research Center, “Two-Thirds of Americans Expect Presidential Election Will Be Disrupted by COVID-19,” April 28, 2020, https://www.people-press.org/2020/04/28/two-thirds-of-americans-expect-presidential-election-will-be-disrupted-by-covid-19/ (accessed September 9, 2020).

## Supplementary material

For supplementary material accompanying this paper visit https://doi.org/10.1017/S1743923X20000641. click here to view supplementary material
