# Supplementary material for: Voting in a Pandemic: Explaining Variation in Support for Absentee Ballots for All
Source: Politics & Gender. 2020 Dec;16(4):1093–100. doi: 10.1017/S1743923X20000641 (PMC7853733; doi:10.1017/S1743923X20000641)
Supplement: Supplementary file 1 [file S1743923X20000641sup001.docx]

**Online Appendix**

*Table 1* (full model). Multinomial logistic regression coefficients for the probability for support for absentee ballots using restrict as the baseline comparison using the National Panel Study of COVID-19

|  | ***Full Model*** | | ***Men*** | | ***Women*** | | |
| --- | --- | --- | --- | --- | --- | --- | --- |
|  | Allow | Depends | Allow | Depends | Allow | Depends | |
| Variables | Odds Ratio | Odds Ratio | Odds Ratio | Odds Ratio | Odds Ratio | Odds Ratio | |
|  |  | | | | | | |
| Female | 1.258* | 0.499*** |  |  |  |  |  |
|  | (0.121) | (0.178) |  |  |  |  |  |
| Corona worry | 1.033 | 0.612*** | 1.075 | 0.535** | 0.990 | 0.740 | |
|  | (0.131) | (0.177) | (0.209) | (0.260) | (0.166) | (0.235) | |
| Corona network risk | 1.509** | 0.828 | 1.360 | 0.649 | 1.655** | 0.863 | |
| Reference category: White | (0.189) | (0.341) | (0.284) | (0.500) | (0.250) | (0.456) | |
| Latino | 0.832 | 0.508* | 0.857 | 0.736 | 0.789 | 0.182** | |
|  | (0.201) | (0.410) |  |  |  |  | |
| Black | 0.924 | 0.635 | 1.293 | 0.975 | 0.553** | 0.115** | |
|  | (0.189) | (0.378) | (0.267) | (0.461) | (0.258) | (1.051) | |
| Asian | 0.439*** | 0.521 | 0.443** | 0.804 | 0.391*** | 0.242 | |
|  | (0.270) | (0.579) | (0.406) | (0.707) | (0.364) | (1.044) | |
| Education | 1.180*** | 0.994 | 1.133 | 0.895 | 1.259*** | 1.157 | |
|  | (0.049) | (0.070) | (0.080) | (0.109) | (0.061) | (0.098) | |
| Age | 1.017*** | 1.024*** | 1.019** | 1.019* | 1.015*** | 1.032*** | |
|  | (0.005) | (0.008) | (0.008) | (0.011) | (0.006) | (0.011) | |
| Family income | 1.041* | 1.051 | 1.033 | 1.109** | 1.053* | 0.990 | |
|  | (0.023) | (0.034) | (0.036) | (0.050) | (0.029) | (0.046) | |
| Reference: Republican |  |  |  |  |  |  | |
| Democrat | 1.546*** | 0.557** | 1.122 | 0.426** | 2.082*** | 0.715 | |
|  | (0.169) | (0.262) | (0.253) | (0.381) | (0.215) | (0.367) | |
| Independent | 1.121 | 1.106 | 0.822 | 1.137 | 1.425 | 0.910 | |
|  | (0.171) | (0.225) | (0.264) | (0.326) | (0.217) | (0.310) | |
| Political ideology (Lib.– Cons.) | 0.735*** | 1.529*** | 0.730*** | 1.617*** | 0.720*** | 1.311** | |
|  | (0.063) | (0.094) | (0.093) | (0.131) | (0.081) | (0.135) | |
| Reference category: Single no kids |  |  |  |  |  |  | |
| Single with kids | 0.816 | 1.019 | 0.631 | 1.402 | 0.908 | 0.787 | |
|  | (0.285) | (0.661) | (0.501) | (0.856) | (0.354) | (1.139) | |
| Married no kids | 0.881 | 1.374 | 1.150 | 1.332 | 0.674 | 1.834 | |
|  | (0.203) | (0.318) | (0.302) | (0.426) | (0.267) | (0.486) | |
| Married with kids | 0.637** | 1.030 | 0.582* | 1.184 | 0.738 | 1.426 | |
|  | (0.204) | (0.334) | (0.300) | (0.436) | (0.281) | (0.553) | |
| Divorced no kids | 0.652* | 0.979 | 0.770 | 0.861 | 0.568** | 1.239 | |
|  | (0.231) | (0.325) | (0.375) | (0.445) | (0.287) | (0.495) | |
| All else | 0.727* | 1.190 | 0.838 | 1.631 | 0.634* | 1.043 | |
|  | (0.187) | (0.313) | (0.289) | (0.409) | (0.247) | (0.477) | |
| Reference category: Employed |  |  |  |  |  |  | |
| Unemployed due to coronavirus | 1.019 | 1.260 | 0.884 | 1.547 | 1.151 | 1.015 | |
|  | (0.182) | (0.310) | (0.306) | (0.454) | (0.221) | (0.395) | |
| Unemployed not looking for work | 1.328* | 1.854** | 1.140 | 3.011*** | 1.520** | 1.101 | |
|  | (0.153) | (0.247) | (0.252) | (0.354) | (0.193) | (0.290) | |
| Constant | 0.326*** | 0.032*** | 0.400 | 0.037*** | 0.355** | 0.019*** | |
|  | (0.421) | (0.660) | (0.665) | (1.009) | (0.500) | (0.847) | |
| Observations | 1,892 | 1,892 | 776 | 776 | 1,116 | 1,116 | |
| Adjusted *R*^2^ | 0.123 | 0.123 | 0.133 | 0.133 | 0.126 | 0.126 | |
| Robust standard errors in parentheses, using national weights. | |  |  |  |  |  | |
| *** *p* < .01; ** *p* < .05; * *p* < .1. |  |  |  |  |  |  | |
